# The potential influence of follicle diameter on natural cycle in vitro fertilization among women with diminished ovarian reserve: a retrospective cohort study

**DOI:** 10.1186/s13048-023-01281-4

**Published:** 2023-09-20

**Authors:** Tian Tian, Yu Li, Jiaxin Lv, Lixue Chen, Yuanyuan Wang, Rui Yang, Ping Liu, Rong Li, Jie Qiao

**Affiliations:** 1 Center for Reproductive Medicine, Department of Obstetrics and Gynecology, Peking University Third Hospital, Beijing, China; 2 National Clinical Research Center for Obstetrics and Gynecology, Peking University Third Hospital, No. 49 Hua Yuan Bei Road, Hai Dian District, Beijing, 100191 China; 3 Key Laboratory of Assisted Reproduction (Peking University), Ministry of Education, Beijing, China; 4 Beijing Key Laboratory of Reproductive Endocrinology and Assisted Reproductive Technology, Peking University Third Hospital), Beijing, China; 5 Aviation General Hospital, Beijing, China; 6 Beijing Advanced Innovation Center for Genomics, Beijing, China; 7 Peking-Tsinghua Center for Life Sciences, Peking University, Beijing, China

**Keywords:** Natural-cycle, In vitro fertilization, Follicle diameter, 2PN fertilization, Pregnancy outcome

## Abstract

**Background:**

Natural cycle- in vitro fertilization (NC-IVF) is particularly recommended for women with decreased ovarian reserve (DOR) or poor response to controlled ovarian hyperstimulation. In these cases, it can be challenging to determine the optimal timing for a trigger, and follicles of varying sizes are typically obtained. The influence of follicular size on IVF outcomes in women with DOR remains uncertain. This study aims to investigate the association between different follicular sizes and NC-IVF outcomes in women with DOR.

**Methods:**

A retrospective cohort study involving 477 NC-IVF cycles from 2015 to 2021 was conducted at one of the largest reproductive medical centers in China. Follicular growth was monitored using transvaginal ultrasonography, and the follicles were categorized into three groups based on their diameters:12–15 mm; 16–17 mm and ≥ 18 mm. Laboratory outcomes were evaluated, including the number of canceled cycles, number of oocytes retrieved, 2PN fertilization, embryo and good-quality embryo, fresh embryo transfers, and frozen embryo. Additionally, clinical outcomes, such as the rates of biochemical pregnancy, clinical pregnancy, ongoing pregnancy, and live birth, were investigated and compared among the different follicular size groups.

**Results:**

A total of 68 cycles with follicles sizes of 12-15 mm, 171 cycles with follicles sizes of 16-17 mm, and 236 cycles with follicles sizes ≥ 18 mm were included in this study. The basic characteristics, including female age, male age, infertility duration, infertility type, and parity, were comparable among the groups. The rate of cycle cancellation in the 12–15 mm group (27.9%) was higher compared to the other two groups. The 2PN fertilization rate for follicles with a diameter of 16-17 mm (75.0%) was higher than that of follicles with a diameter of 12-15 mm (61.3%) and ≥ 18 mm (56.6%) (*P* = 0.031). Other clinical outcomes, such as the number of oocytes retrieved, good-quality embryos, fresh embryo transfers, and frozen embryos, did not show significant differences between groups. Further analysis revealed no significant difference in the rates of clinical pregnancy, ongoing pregnancy, and live birth rate among the three groups.

**Conclusions:**

This study indicates that in women with DOR undergoing NC-IVF, if a premature LH surge occurs and small follicles are retrieved, these follicles can still be used in subsequent treatment and provide a comparable chance of clinical pregnancy to normal-sized follicles. These findings have important implications for guiding NC-IVF treatment in patients with severe DOR.

**Trial registration number:**

N/A.

## Introduction

In vitro fertilization (IVF) is a vital procedure for couples experiencing infertility and desiring to have a baby. Recently, natural cycle IVF (NC-IVF) has been increasingly performed in many countries [14]. NC-IVF is based on the natural recruitment and selection of follicles without the use of controlled ovarian hyperstimulation (COH) and with an unsupported luteal phase. Compared to standard IVF-ET with COH, NC-IVF is generally considered to be friendly due to its lower cost, repeatability without embryo selection and cryopreservation [4, 11], and the lighter psychological burden on patients [15]. Several studies revealed that compared with IVF with ovarian stimulations, NC-IVF had better pregnancy outcomes. For instance, one study reported a higher embryo implantation rate in advanced-age poor responders undergoing NC-IVF [1], while another study found a lower risk of low birth weight in infants born through NC-IVF [9].

NC-IVF is particularly recommended for women with an extremely low ovarian reserve or those who have a poor response to COH and are not suitable for egg donation [3]. However, since NC-IVF does not involve the use of ovulation-inducing drugs, the number of achievable oocytes in a single cycle is typically limited to just one. This can pose a challenge for patients with DOR and a reduced ability to induce ovulation [7].

Follicular growth monitoring is an essential step in NC-IVF. The initial follicular growth assessment typically occurs between the 10th and 12th cycle days of the 28-day cycle. When the follicular diameter was usually expected to reach at least 16 mm and estradiol (E2) concentration was expected to be 700 pmol/L, 5000 IU human chorionic gonadotropin (HCG) was administered subcutaneously 36 h prior to oocyte retrieval. However, in the clinical practice of NC-IVF, accurately determining the exact timing of follicular growth is often challenging, resulting in uneven sizes of retrieved follicles. While a limited number of studies have explored the association between follicular size and IVF outcomes, focusing on women with normal ovarian reserve [2, 5, 6], the influence of different follicular sizes on IVF outcomes in women with DOR remains uncertain.

Therefore, in this study, we conducted a retrospective cohort study to investigate the effects of follicular size on both laboratory and pregnancy outcomes in women with DOR. We aim to provide valuable insights and guidance for the clinical monitoring and optimal timing of oocyte retrieval in the NC-IVF procedure and provide implications for guiding NC-IVF treatment in patients with severe DOR.

## Materials and methods

### Study participants

**Fig. 1:**
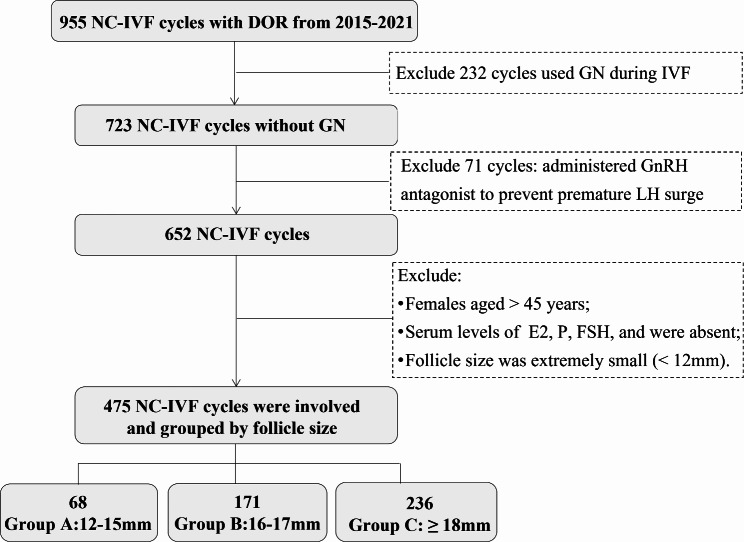
The flow chart of participants selection

The retrospective cohort study initially included 955 participants who had DOR and underwent NC-IVF cycles between 2015 and 2021 at the Center for Reproductive Medicine, Peking University Third Hospital, one of the largest reproductive health centers in China. Diagnoses of DOR in this study are made by clinical physicians based on the following criteria: i) summation of bilateral antral follicle count (AFC) ≤ 7 and the serum anti-müllerian hormone (AMH) level ≤ 1.1 ng/mL. The assessment of AFC and AMH was described in detail in our previous publication [16]. Participants were further excluded based on the following criteria: (1) Cycles that used gonadotropin (Gn) during IVF; (2) Cycles that administered gonadotrophin-releasing hormone (GnRH) antagonist or other ovulation induction drugs (including clomiphene, letrozole and so on) to prevent premature luteinizing hormone (LH) surge; (3) Females aged > 45 years; 3) Serum concentrations of estradiol (E2), progesterone (P), follicle-stimulating hormone (FSH), and LH in serum were absent; (4) The follicle diameter was extremely small (< 12 mm). Ultimately, a total of 475 cycles were included in the analysis **(**Fig. 1**).**

This study was approved by the Ethics Committee of Peking University Third Hospital (No. IRB00006761-M2020004).

### The monitoring of the follicles

Follicular growth was monitored using transvaginal ultrasonography on the eighth day of the menstruation cycle. During the subsequent regular control visits, the levels of E2, P, and LH were regularly measured, and the follicle and endometrium thickness was assessed by ultrasound in millimeters (mm). Based on the monitored size of the follicle and serum levels of LH, E2, and P, the timing of the hCG injection was decided. Patients who demonstrated an LH surge also underwent triggerring. Usually when the dominant follicle reached a diameter of 16–18 mm, an hCG trigger was administered, and oocyte retrieval was performed 36–38 h later. In cases where patients exhibited monitored follicles sized between 12 and 15 mm, an earlier trigger or oocyte retrieval could be performed if a premature LH surge or a history of small follicle ovulation was observed, leading to the retrieval of 12-15 mm follicles. Follicles were retrieved by aspiration by employing an ultrasonically guided vaginal probe with or without the sedation or anesthesia. Follicles were classified into three groups based on their diameters measured by the last ultrasound. Group A: mean diameter of 12–15 mm. Group B: mean diameter of 16–17 mm; Group C: mean diameter of ≥ 18 mm. Prior to follicular aspiration, the oocyte retrieval was canceled if no follicle was observed during the B-ultrasound examination. All cycles included in this study involved only one follicle.

### The outcomes variables

The insemination procedures were conducted following the standard ICSI/IVF protocols. After oocyte examination for normal fertilization, the oocytes were cultured in an IVF medium for 16 to 20 h. Fertilization was defined as the presence of two pronuclei (2PN) and two polar bodies. The following outcomes were observed and recorded in this study:

#### Laboratory outcomes

The primary laboratory outcomes included the number of cycles with oocytes retrieved and fertilization in each group. Cycle cancellation refers to oocyte retrieval was canceled in some cases due to premature ovulation and subsequent follicle rupture. It should be noted that cycles in which no oocytes were obtained during retrieval are not included in the definition of cycle cancellation. Embryo quality was assessed. On day 3, embryos that reached the 6-cell stage with ≤ 20% fragmentation were classified as good-quality embryos.

#### Clinical outcomes

The clinical outcomes encompassed biochemical pregnancy, ongoing pregnancy, and live birth. Biochemical pregnancy was defined as hCG levels of 25 IU/L that spontaneously resolved without ultrasound evidence of either an intrauterine or an ectopic pregnancy 12–14 days after embryo transfer. Clinical pregnancy was defined as the detection of a gestational sac by ultrasonography. Ongoing pregnancy was defined as the visualization of an intrauterine sac with an embryonic pole demonstrating cardiac activity after 10–12 gestational weeks. Live birth was defined as the delivery of live babies beyond 28 weeks of pregnancy or with a birth weight > 1000 g [17].

### Sample size estimation

The sample size estimation was mainly based on the two primary outcomes, oocyte retrieval and fertilization rate. It was estimated that the oocyte retrieval rate in 16-17 mm was about 70%, while the group of follicular size < 15 mm might have a reduction of around 15–20% in the retrieval rate. With a power of 0.8, and the sample size ratio of 2, the estimated sample size for each group was 47 vs. 94. Regarding the fertilization rate, it was assumed to be around 75% in the 16-17 mm group, while the rate was expected to decrease to 60% in the < 15 mm group. The sample size required was 60 vs. 120, with a power of 80%. The sample size was deemed sufficient to detect differences in the primary outcomes between the groups.

### Statistical analysis

Given the non-normally distribution of the continuous data in this study, the median and interquartile ranges (IQR) were used to represent the continuous variables. The Kruskal-Wallis test was employed to compare continuous variables among different groups, and subsequent multiple comparisons were conducted between every two groups. Categorical variables were described by the number of cases and percentages and were compared with the Chi-squared or Fisher’s exact test, as appropriate. The tests were considered statistically significant when a two-sided *P* value was less than 0.05. All analyses were performed using SPSS version 24.0.

## Results

### The basic characteristics of the study population

As Fig. 1 shows, between 2015 and 2021, a total of 475 NC-IVF cycles were involved, including 68 cycles with follicles size of 12-15 mm, 171 cycles with follicles size of 16–17 mm, and 236 cycles with follicles size ≥ 18 mm. Comparing the different follicle diameter groups revealed no significant differences in female age, infertility years, the frequencies of IVF history, primary infertility, parities, or insemination methods. Furthermore, the basic levels of FSH, E2, and LH did not show any significant difference between groups. The baseline level of AMH was higher in the follicle diameter ≥ 18 mm group [0.23 (0.05–0.46) ng/ml], and significantly lower in the 12-15 mm [0.16 (0.05–0.34 ng/ml]. Additionally, 140 cycles were duplicate cases, with percentages of repeated cycles in the 12-15 mm, 16-17 mm and > 18 mm groups of 29.4%, 33.9% and 26.3%, respectively, showing no significant difference between groups (Table 1).

**Table 1:** Basic characteristics of participants

Follicle size		12-15 mm (N = 68)	16-17 mm (N = 171)	≥ 18 mm (N = 236)	H/χ^2^	*P*
**Female age**		37.5 (33.0-41.8)	38.0 (32.0–42.0)	38.0 (33.0–42.0)	1.708	0.426
**Female BMI**		21.7 (20.4–23.3)	21.5 (20.0-23.3)	22.3 (20.2–24.8)	7.712	0.021
**Male age**		37.0 (31.3–41.7)	38.0 (35.0–44.0)	39.0 (34.0–44.0)	4.128	0.127
**Male BMI**		26.0 (23.7–27.7)	26.0 (23.0-27.7)	25.4 (22.8–28.7)	7.827	0.020
**Infertility duration**		2.0 (2.0–6.0)	3.0 (2.0–7.0)	4.0 (2.0-7.3)	0.963	0.618
**History of IVF**	No	18 (27.9)	62 (36.0)	99 (41.9)	4.763	0.095
	Yes	49 (72.1)	110 (64.0)	137 (58.1)		
**Infertility type**	Secondary	32 (47.1)	83 (48.3)	109 (46.2)	0.171	0.918
	Primary	36 (52.9)	89 (51.7)	127 (53.8)		
**Parity**	0	40 (58.8)	96 (56.1)	134 (56.8)	1.124	0.890
	1	16 (23.5)	35 (20.5)	48 (20.3)		
	≥ 2	12 (17.6)	40 (23.4)	54 (22.9)		
**Basic level of FSH**	(mIU/mL)	10.05 (7.22–16.67)	11.15 (7.03–16.62)	11.15 (7.94–17.63)	0.322	0.851
**Basic level of E2**	*(pmol*/L)	158.82 (91.75-236.75)	167.5 (107.75-252.25)	150.0 (93.0-199.0)	5.405	0.067
**Basic level of LH**	(mIU/mL)	3.80 (2.46–6.55)	2.81 (4.11–6.53)	4.20 (2.49–6.44)	1.832	0.400
**AMH**	(ng/mL)	0.16 (0.05–0.34)	0.26 (0.11–0.62)	0.23 (0.05–0.46)	6.981	0.030
**Duplicate cycles**	**No**	48 (70.6)	113 (66.1)	174 (73.7)	2.790	0.251
	**Yes**	20 (29.4)	58 (33.9)	62 (26.3)		

Note: Continuous data are presented as median (IQR), while categorical variables were shown as numbers (percentages). ^a^: represents that the patients did not undergo insemination owing to no proper oocytes being obtained

### Laboratory outcomes

Serum levels of sexual hormones were measured before egg retrieval. Table 2 illustrates that the progesterone level did not significantly vary among the groups. However, there was a significant difference in E2 between the three groups. The cycle cancellation rate in the 12-15 mm group was 27.9%, significantly higher than that in the 16-17 mm and ≥ 18 mm groups. The frequencies of cycles that retrieved oocytes and obtained embryos were comparable across the three groups. In addition, the rates of fresh embryo transfer and frozen embryos between groups showed no significant difference. Notably, the 2PN fertilization rates differed in the three groups (all *Ps* > 0.05). Multiple comparisons indicated that the 2PN fertilization rate in follicle diameter ≥ 18 mm group (60.0%) was significantly lower than in the 16-17 mm group (75.5%, *P* < 0.05). Although no statistical difference was shown, the rate of embryos of good quality was slightly lower in the 12-15 mm group compared to the other two groups (36.4% vs. 36.8% vs. 38.5%).

**Table 2:** The laboratory markers in different groups

	12-15 mm (N = 68)	16-17 mm (N = 171)	≥ 18 mm (N = 236)	H/χ^2^	P
**E2/LH/P** ^†^					
E2 (*pmol*/L)	790.0 (593.0-965.0)	918.0 (683.0-1124.5)	921.0 (657.0-1263.0)	5.908	0.050
LH (mIU/mL)	13.25 (7.39–22.97)	10.9 (6.525–23.9)	8.72 (5.43–13.45)	16.158	0.000
P (nmol/L)	1.24 (0.81–1.88)	1.14 (0.85–1.745)	1.16 (0.8–1.73)	1.178	0.555
**No. of canceled cycle** ^†^	20/68 (27.9)	21/171 (12.3)^*a*^	30/236 (12.7)^*c*^	11.027	0.004
**No. of oocytes retrieved** ^§^	33/48 (64.7)	106/150 (70.7)	130/206 (67.8)	0.718	0.698
**Insemination method**					
**ICSI**	9 (27.3)	17 (16.0)	27 (20.8)	2.189	0.335
**IVF-ET**	24 (72.7)	89 (84.0)	103 (79.2)		
**No. of 2PN fertilization** ^¶^	21/33 (63.6)	80/106 (75.5)	78/130 (60.0) ^*b*^	6.421	0.040
**No. of embryo** ^¶^	13/33 (33.3)	42/106 (37.5)	55/130 (38.9)	0.896	0.639
**No. of good-quality embryo** ^¶^	12/33 (36.4)	39/106 (36.8)	50/130 (38.5)	0.094	0.955
**No. of fresh embryo transfers** ^&^	9/13 (69.2)	29/42 (69.0)	35/55 (32.6)	0.662	0.832
**No. of frozen embryo** ^&^	4/13 (30.8)	13/42 (31.0)	20/55 (33.6)	0.662	0.832

Note: Continuous data are presented as median (IQR), while categorical variables were shown as numbers (percentages). The Chi-Square or Kruskal-Wallis tests were performed to examine the difference between the three groups. Multiple comparisons between every two groups were further performed. ^*a*^ represents there is a significant difference between group ≤ 15 mm and 16-17 mm; ^*b*^ represents there is a significant difference between group 16-17 mm and ≥ 18 mm and ^*c*^ represents a significant difference between group ≤ 15 mm and ≥ 18 mm

† denotes that the denominator was the total number of cycles in each group

§ indicates that the denominator excluded any canceled cycles

¶ signifies that the denominator was the number of cycles from which oocytes were obtained

& represents the denominator as the number of obtained embryos in each group

### Pregnancy outcomes

The frequencies of pregnancy outcomes are presented in Table 3. Among the 12-15 mm group, 3 out of 13 women (23.1%) achieved clinical pregnancy, which was higher than the rates in the 16-17 mm group (8/42, 19.0%) and ≥ 18 mm group (12/55, 21.8%). The rates of ongoing pregnancy were 2/13 (15.4%), 5/42 (11.9%), and 9/55 (16.4%) in the 12-15 mm group, 16-17 mm group, and ≥ 18 mm group, respectively. Similarly, the rates of live birth in the 12-15 mm group (15.4%) were higher than in 16-17 mm (9.5%) and ≥ 18 mm (12.7%). However, due to the small sample size, these pregnancy outcome did not show any statistical differences between the groups.

**Table 3:** Pregnancy outcomes in different groups

	12-15 mm	16-17 mm	≥ 18 mm	χ^2^	*P*
**Biochemical pregnancy**	3/13 (23.1)	8/42 (19.0)	12/55 (21.8)	0.273	0.895
**Clinical pregnancy**	3/13 (23.1)	6/42 (14.3)	10/55 (18.2)	0.789	0.730
**Ongoing pregnancy**	2/13 (15.4)	5/42 (11.9)	9/55 (16.4)	0.497	0.735
**Live birth**	2/13 (15.4)	4/42 (9.5)	7/55 (12.7)	0.649	0.765

Note: The denominator was the number of obtained embryos

## Discussion

In ART treatment, NC-IVF is particularly recommended for women with a very low ovarian reserve or poor response to COH due to its lower cost, repeatability, and lack of requirement for embryo selection and cryopreservation. However, in NC-IVF, the retrieval process may be canceled if there is a premature luteinizing hormone (LH) surge. In clinical practice, it is often challenging to accurately determine the timing of follicular growth, resulting in uneven sizes of the retrieved follicles. A limited study has investigated whether the size of retrieved follicles in NC-IVF cycles, particularly in women with DOR, influences IVF outcomes. Therefore, in this retrospective cohort population study, we focused on women with an extremely low ovarian reserve, and aimed to provide new evidence regarding the relationship between follicle diameter and laboratory and pregnancy outcomes in NC-IVF cycles. We found that the 2PN fertilization rate was affected by follicle diameter, while other outcomes did not demonstrate significant differences among the different follicle diameter groups. These findings provide new clues and help clinicians make decisions during the NC-IVF treatment.

Recently, one study reported that in NC-IVF, non-dominant small follicle puncture yielded comparable numbers of metaphase II oocytes and live births with large dominant follicle puncture, indicating that non-dominant small follicles are a promising supplementary source of mature oocytes for NC-IVF [12]. Another study investigated the outcome parameters based on follicle diameter per aspiration and found that follicles with a diameter of 14–15 and 21–22 mm had lower oocyte collection rates compared to follicles of 16–20 mm, leading to lower rates of mature oocytes and embryos, although the differences were not significant [6]. However, these studies mainly focused on women with normal ovarian reserve. In this study, we retrieved some small follicles in the clinic at the time when we observed the abnormal LH level among DOR women. We found that in DOR women, compared to 16–17 mm, follicles sized 12–15 mm had a higher rate of cycle cancellation and slightly lower rates of 2PN fertilization and good-quality embryos, although these differences were not statistically significant. Neither did the other pregnancy outcomes, including clinical pregnancy rates, ongoing pregnancy, and live birth, show any significant difference between small and normal-sized follicles. These findings suggest that in NC-IVF cycles for women with extreme DOR, 12–15 mm follicles still have a similar chance of achieving successful pregnancy compared to women with normal-sized follicles. Additionally, we observed that despite the high cancellation rate in the 12–15 mm group, the rate of clinical pregnancy was high, indicating that women who experience an early LH peak may still proceed with the IVF process. However, further validation is needed due to the small sample size of our study.

In IVF/ICSI with COH, whether larger follicles could improve IVF outcomes has been inconclusive. One study reported that follicles larger than 18 mm at retrieval have consistently mature oocytes with a higher fertilization rate [10]. Another investigation showed a tendency of increased fertilization rate and rate of good quality embryos from small to large follicle groups, although not statistically significant [13]. On the contrary, other studies reported that large follicles (e.g.,≥ 17 mm) in IVF may lead to follicular luteinization, resulting in a lower ongoing pregnancy rate [8]. Our current study found that large-size follicles (≥ 18 mm) did not improve the laboratory and pregnancy outcomes in NC-IVF. On the contrary, the 2PN fertilization rate in the ≥ 18 mm follicle group was significantly lower than in the 16-17 mm group. These findings suggest that for women without a premature rise in LH, a follicle size of 16–17 mm may be a more suitable endpoint for follicle monitoring in NC-IVF, rather than 18 mm.

During NC-IVF treatment, every step can cause follicle or egg loss, including cycle cancellation, fertilization failure, etc. Therefore, in this study, even in the normal-sized follicle group, the final rates of clinical pregnancy and live birth were low, ranging from 13.3 to 18.2%. Therefore, for women with a normal response to COH, we recommend a stimulation cycle with IVF/ICSI instead of NC-IVF to improve the pregnancy rate. However, for women with extreme DOR or POR, considering the low rates of clinical pregnancy and live birth, we would recommend repeating several cycles of NC-IVF to accumulate and transfer multiple embryos, thus increasing the chances of IVF success.

To the best of our knowledge, our study, for the first time, investigated the associations between follicle diameters and outcomes in NC-IVF for women with DOR. We strictly used including criteria to control the potential confounders by GnRH antagonist or GN administration. Our findings demonstrate that small follicles do not significantly impact IVF outcomes, suggesting that women with an extremely low ovarian reserve can continue the IVF process even with small follicles, and they may have comparable chances of success to women with relatively larger follicles. Our study provides essential guidance for the IVF treatment of patients with severe DOR. However, some limitations require to be addressed before any potential extrapolation of the findings. First, the proportion of the NC-IVF cycle is still tiny (about 1–2% in all cycles) in the clinic, and we selected the women with DOR from these NC-IVF cycles, which further reduced the sample size. Second, the pregnancy/live birth rates were meager in DOR women who underwent NC-IVF cycles. Although we included all NC-IVF cycles that met the inclusive criteria from 2015 to 2021 in our center, the sample size, especially in the analysis of pregnancy outcomes, was evitably small. Thus, we cannot exclude the possibility of false negatives in our results. The small sample size also makes it challenging to perform further association analysis using a regression approach (e.g. number of live births in small follicles was 2). We will collect more samples and validate the findings of this study in future research. Third, the clinical protocol information was obtained from medical records, which did not provide detailed information regarding the selection criteria for fresh and frozen embryo transfers, making it difficult for us to ascertain the exact reasons behind the choices made. In addition, development stage of oocytes were not evaluated in most cycles because of routine IVF in this study.

## Conclusion

In this retrospective population study, we found that among women with DOR who underwent NC-IVF treatment, the outcomes of the small follicle (≤ 15 mm) were comparable to those of normal-sized follicles. However, follicles with a diameter of ≥ 18 mm exhibited a lower 2PN fertilization rate compared to 16–17 mm follicles. Our study indicates that if the premature rise in LH was shown and small follicles were obtained in NC-IVF, these follicles can still be considered for subsequent treatment, providing patients with a chance of achieving clinical pregnancy.

## Data Availability

The data underlying this article will be shared upon reasonable request to the corresponding author.
